# Pseudogaucher cells obscuring multiple myeloma: a case report

**DOI:** 10.1186/1757-1626-2-9147

**Published:** 2009-12-04

**Authors:** Vijay Saroha, Parul Gupta, Meeta Singh, Tejinder Singh

**Affiliations:** 1 Department of Pathology, Maulana Azad Medical College and Lok Nayak Hospital, Bahadur Shah Zafar Marg, New Delhi 110002, India

## Abstract

Gaucher-like or pseudo-Gaucher cells have been noted in a variety of conditions including acute lymphoblastic leukemia, Hodgkin's disease, thalassemia, and multiple myeloma. They have an eccentric, lobulated nucleus, foamy cytoplasm but lack the tubular inclusions seen in Gaucher cells. The pseudo-Gaucher cells have distinct appearances on electron microscopy which distinguish them from true Gaucher cells.

Increased pseudo-Gaucher cells probably reflects the increased load of leukocyte membrane derived glucosylceramide presented to macrophages under conditions of high cell turnover when the normal pathways for its removal may be saturated.

We present a case of a 72-year-old Indian Aryan female, in which the bone marrow contained sheets of histiocytes with features mimicking gaucher cells. These pseudo-Gaucher cells obscured neoplastic plasma cells causing diagnostic difficulty.

## Case presentation

A 72-year-old Indian Aryan female known case of controlled type II diabetes mellitus and osteoarthritis presented with easy fatigability and breathlessness for 1 month. On examination she had severe pallor with no lymphadenopathy or splenomegaly. The hemogram showed Hb of 5.3 g/dl, TLC of 3320/cumm and platelet count of 45000/cumm. The red cells showed Rouleaux formation with normocytic normochromic RBCs. ESR was 100 mm in first hour by Westergren's method. Thus, bone marrow aspirate and biopsy were performed.

**Figure 1:**
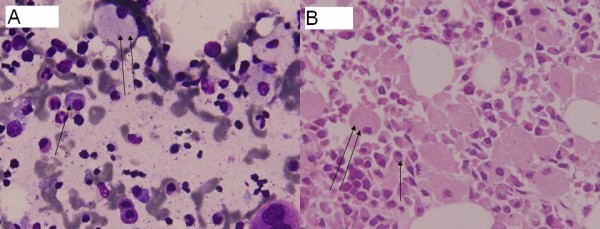
**(a) Bone marrow aspirate smears (Giemsa 400×) and (b) bone marrow biopsy (H&E 400×) showing plasma cells (single arrow) intermixed in between sheets of histiocytes; pseudogaucher cells (double arrow)**.

**Figure 2:**
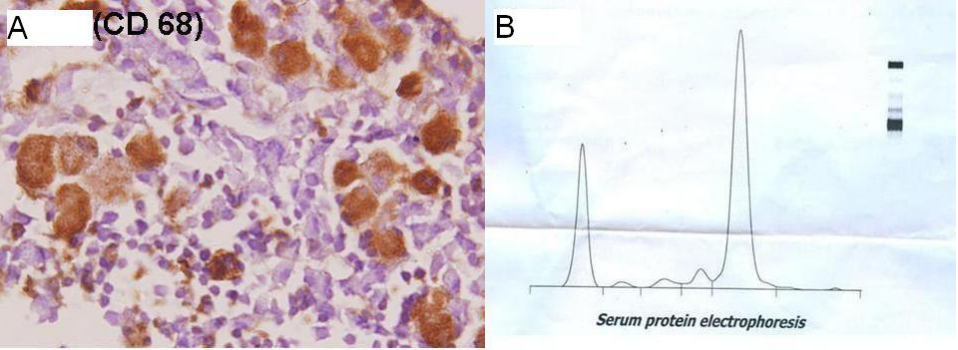
**(a) Pseudogaucher cells positive for CD68**. (b) M band on Serum electrophoresis.

The bone marrow showed sheets of large cells with abundant cytoplasm and a small eccentric pyknotic nucleus. Scattered among these were plasma cells which were obscured by these sheets of histiocytes (Figure 1a and Figure 1b). The erythroid and myeloid series were normal although megakaryocytes were decreased. These large cells were positive for CD68 confirming their histiocytic nature (Figure 2a). These histiocytes were also periodic acid-Schiff positive, resistant to diastase digestion. Immunohistochemically the plasma cells were monoclonal with lambda positivity. No clinical evidence of inherited Gaucher's disease like organomegaly was found in this patient.

Based on these findings serum electrophoresis was done which revealed M band (Figure 2b). X ray skull showed lytic bone lesions.

Thus a diagnosis of multiple myeloma associated with a prominent pseudo-Gaucher histiocytic response was made.

## Discussion

Gaucher-like or pseudo-Gaucher cells have been seen in a variety of conditions such as acute lymphoblastic leukemia, Hodgkin's disease, thalassemia, and multiple myeloma [1].

They are considered to be marrow macrophages seen in conditions associated with high cell turnover [2].

While plasma cells with atypical morphology, such as the mott cells, the flame cells, the morula cells, and the signet ring-like cells, can be found frequently in the marrow of patients with multiple myeloma, cells with Gaucher-like appearance has been only occasionally detected [3].

The pseudo-Gaucher cells are similar in many ways to true Gaucher on light microscopic examination but certain distinguishing features exist. Gaucher cells show diffuse iron staining, whereas pseudo-Gaucher cells are generally negative. Gaucher cells are typically large cells measuring 50 to 60 μm in diameter with a small eccentric nucleus and fibrillary cytoplasm [4]. On electron microscopy, Gaucher cells contain tubular cytoplasmic inclusions, which are absent in pseudo-Gaucher cells [5] that instead contain crystals. These are derived from increased load of leukocyte membrane derived glucosyl-ceramide presented to macrophages under conditions of high cell turnover [2]. Alternatively, Scullin DC Jr et al suggested that these crystals are synthesized in plasma cells and the marrow macrophages acquire them by phagocytosis of crystals released from necrotic plasma cells [5].

Gaucher's disease is a group of autosomal recessive disorders, resulting from the mutations at the glucocerebrosidase locus on chromosome 1q21. As a result, glucocerebroside accumulates in the phagocytic cells of the body. Its diagnosis can be made by measuring the leukocyte glucosidase activity in the presence of organomegaly, cytopenias and typical Gaucher cells [6]. No organomegaly was present in our case.

Gaucher-like cells in bone marrow have also been reported in cases of atypical mycobacterial infection especially in immunocompromised patients [7]. Therefore, stains for acid fast bacilli (AFB) should be performed which was negative in our case.

In our case of multiple myeloma pseudo-Gaucher cells in large numbers obscured neoplastic plasma cells. Immunohistochemical positivity for lambda in plasma cells, M band on serum electrophoresis and lytic lesions on X-ray skull clinched the final diagnosis.

We are reporting this case to make the reporting pathologist aware of this rare association to avoid misdiagnosis and also to highlight that the presence of Pseudogaucher cells in bone marrow should not be overlooked as they might be obscuring an underlying primary pathology. Awareness of possible associations, appropriate immunohistochemistry along with relevant additional investigations based on clinical findings is necessary for final diagnosis.

## Consent

Written informed consent was obtained from the patient for publication of this case report and accompanying images. A copy of the written consent is available for review by the Editor-in-Chief of this journal.

## Competing interests

The authors declare that they have no competing interests.

## Authors' contributions

VS analyzed and interpreted the patient data regarding the hematological disease. PG, TS gave major contributions in writing the manuscript. MS helped in putting immunohistochemical markers. All authors have read and approved the final manuscript.
